# Early Estimates of Seasonal Influenza Vaccine Effectiveness — United States, January 2015

**Published:** 2015-01-16

**Authors:** Brendan Flannery, Jessie Clippard, Richard K. Zimmerman, Mary Patricia Nowalk, Michael L. Jackson, Lisa A. Jackson, Arnold S. Monto, Joshua G. Petrie, Huong Q. McLean, Edward A. Belongia, Manjusha Gaglani, LaShondra Berman, Angie Foust, Wendy Sessions, Swathi N. Thaker, Sarah Spencer, Alicia M. Fry

**Affiliations:** 1 Influenza Division, National Center for Immunization and Respiratory Diseases, CDC; 2 University of Pittsburgh Schools of the Health Sciences and UPMC; 3 Group Health Research Institute; 4 University of Michigan School of Public Health, Department of Epidemiology; 5 Marshfield Clinic Research Foundation; 6 Baylor Scott and White Health, Texas A&M University Health Science Center College of Medicine

In the United States, annual vaccination against seasonal influenza is recommended for all persons aged ≥6 months (1). Each season since 2004–05, CDC has estimated the effectiveness of seasonal influenza vaccine in preventing medically attended acute respiratory illness (ARI) associated with laboratory-confirmed influenza. This season, early estimates of influenza vaccine effectiveness are possible because of widespread, early circulation of influenza viruses. By January 3, 2015, 46 states were experiencing widespread flu activity, with predominance of influenza A (H3N2) viruses (2). This report presents an initial estimate of seasonal influenza vaccine effectiveness at preventing laboratory-confirmed influenza virus infection associated with medically attended ARI based on data from 2,321 children and adults enrolled in the U.S. Influenza Vaccine Effectiveness Network (Flu VE) during November 10, 2014–January 2, 2015. During this period, overall vaccine effectiveness (VE) (adjusted for study site, age, sex, race/ethnicity, self-rated health, and days from illness onset to enrollment) against laboratory-confirmed influenza associated with medically attended ARI was 23% (95% confidence interval [CI] = 8%–36%). Most influenza infections were due to A (H3N2) viruses. This interim VE estimate is relatively low compared with previous seasons when circulating viruses and vaccine viruses were well-matched and likely reflects the fact that more than two-thirds of circulating A (H3N2) viruses are antigenically and genetically different (drifted) from the A (H3N2) vaccine component of 2014–15 Northern Hemisphere seasonal influenza vaccines (2). These early, low VE estimates underscore the need for ongoing influenza prevention and treatment measures. CDC continues to recommend influenza vaccination because the vaccine can still prevent some infections with the currently circulating A (H3N2) viruses as well as other viruses that might circulate later in the season, including influenza B viruses. Even when VE is reduced, vaccination still prevents some illness and serious influenza-related complications, including thousands of hospitalizations and deaths (3). Persons aged ≥6 months who have not yet been vaccinated this season should be vaccinated, including persons who might already have been ill with influenza this season.

CDC always recommends antiviral medications as an adjunct to vaccination, and their potential public health benefit is magnified in the context of reduced vaccine effectiveness. All hospitalized patients and all outpatients at high risk for serious complications from influenza should be treated as soon as possible with a neuraminidase inhibitor medication if influenza is suspected. A CDC health update from January 9, 2015, regarding treatment with antiviral medications is available at http://emergency.cdc.gov/han/han00375.asp. Physicians should not wait for confirmatory influenza laboratory testing, and the decision to use an antiviral medication should not be influenced by patient vaccination status (4). Clinicians should be aware that influenza activity is widespread and influenza should be considered as a possible diagnosis in all patients with acute respiratory illness.

Flu VE methods have been published previously (5). Patients aged ≥6 months were enrolled when seeking outpatient medical care for an ARI with cough at study sites in Michigan, Pennsylvania, Texas, Washington, and Wisconsin.* Study enrollment began once laboratory-confirmed cases of influenza were identified through local surveillance. Trained study staff members reviewed appointment schedules and chief complaints to identify patients with ARI. Patients were eligible for enrollment if they 1) were aged ≥6 months on September 1, 2014, and thus eligible for vaccination; 2) reported an ARI with cough and onset ≤7 days earlier; and 3) had not yet been treated with influenza antiviral medication (e.g., oseltamivir) during this illness. Consenting participants completed an enrollment interview. Nasal and oropharyngeal swabs were collected from each patient and placed together in a single cryovial with viral transport medium. Only nasal swabs were collected for patients aged <2 years. Specimens were tested at Flu VE laboratories using CDC’s real-time reverse transcription–polymerase chain reaction (rRT-PCR) protocol for detection and identification of influenza viruses.

Participants were considered vaccinated if they received ≥1 dose of any seasonal influenza vaccine ≥14 days before illness onset, according to medical records and registries (at the Wisconsin site) or medical records and self-report (at the Michigan, Pennsylvania, Texas, and Washington sites). Vaccine effectiveness was estimated as 100% × (1 − odds ratio [ratio of odds of being vaccinated among outpatients with influenza-positive test results to the odds of being vaccinated among outpatients with influenza-negative test results]); odds ratios were estimated using logistic regression. Estimates were adjusted for study site, age, sex, race/ethnicity, self-rated health, and days from illness onset to enrollment. These early interim VE estimates for the 2014–15 season were based on patients enrolled through January 2, 2015.

Of the 2,321 children and adults with ARI enrolled at the five study sites, 950 (41%) tested positive for influenza virus by rRT-PCR; 916 (96%) of these viruses were influenza A, and 35 (4%) were influenza B (Table 1). The proportion of patients with influenza differed by study site, age, race/ethnicity, and interval from onset to enrollment (Table 1). The proportion vaccinated ranged from 46% to 66% across sites and also differed by age, sex, race/ethnicity, and self-rated health status.

The proportion vaccinated with 2014–15 seasonal influenza vaccine was 49% among patients with influenza compared with 56% among influenza-negative controls (Table 2). After adjusting for study site, age, sex, race/ethnicity, self-rated health, and days from illness onset to enrollment, VE against medically attended ARI attributable to influenza A and B virus infections was 23% (CI = 8%–36%).

Among the 916 infections with influenza A viruses, 842 (92%) viruses were subtyped; 100% of those were influenza A (H3N2) viruses (Table 1). Overall, 24 influenza A (H3N2) viruses from patients enrolled in Flu VE were characterized; eight (33%) were antigenically similar to A/Texas/50/2012, and 16 (67%) were antigenically drifted. The drifted viruses had reduced titers with antiserum produced against A/Texas/50/2012 and were similar to the A/Switzerland/9715293/2013 (H3N2) virus. The adjusted VE for all ages against medically attended ARI caused by influenza A (H3N2) virus infection was 22% (CI = 5%–35%). The adjusted, age-stratified VE point estimates were 26% for persons aged 6 months–17 years, 12% for persons aged 18–49 years, and 14% for persons aged ≥50 years (Table 2). Statistically significant VE was observed only among persons aged 6 months–17 years.

## Discussion

The early onset of the 2014–15 influenza season offered an opportunity to provide an early VE estimate. Overall, the estimate suggests that the 2014–15 influenza vaccine has low effectiveness against circulating influenza A (H3N2) viruses. These early findings are consistent with laboratory data demonstrating that most influenza A (H3N2) viruses circulating in the community are antigenically and genetically different from A/Texas/50/2012, the A (H3N2) component of the 2014–15 Northern Hemisphere influenza vaccine. The predominant A (H3N2) viruses detected through surveillance during the 2014–15 season have been similar to the A/Switzerland/9715293/2013 (H3N2) virus, the H3N2 virus selected for the 2015 Southern Hemisphere influenza vaccine (2). CDC will continue to closely monitor vaccine effectiveness this season, and these estimates might be updated as more data become available. CDC continues to recommend influenza vaccination even when there are drifted viruses circulating because the vaccine can still prevent some infections with the circulating A (H3N2) viruses and might also prevent serious complications requiring hospitalization. Also, vaccine might protect against other influenza viruses that can circulate later. As of early November, 2014, fewer than half of U.S. residents had reported receiving influenza vaccine this season.† Influenza vaccination, even when effectiveness is reduced, can prevent thousands of hospitalizations (3).

The severity and timing of influenza activity during the 2014–15 season has so far been similar to the moderately severe 2012–13 season, the last season when influenza A (H3N2) viruses predominated. Rates of influenza-associated hospitalization so far this season are similar to rates during 2012–13, with highest hospitalization rates among persons aged ≥65 years (2). CDC surveillance through January 3, 2015, shows that the percentage of patient visits to doctors for influenza-like-illness (ILI) this season was almost the same as at the peak of the 2012–13 season (2). For the past 13 seasons, influenza seasons have ranged in duration, with an average of 13 weeks of increased ILI activity. This season, as of the week ending January 3, 2015, influenza activity has been elevated for 7 consecutive weeks, suggesting that the current influenza season might continue for several weeks. Influenza activity might continue to increase, especially in parts of the country that have seen more recent increases in activity and parts of the country that have yet to experience significant influenza activity.

These early VE estimates underscore the need for additional influenza prevention and treatment measures, especially among persons aged ≥65 years, young children, and other persons at higher risk for serious influenza associated complications.§ Influenza antiviral medications should be used as recommended¶ for treatment in patients, regardless of their vaccination status. Antiviral treatment can reduce the duration of illness and reduce complications associated with influenza (4). Antiviral treatment should be used for any patient with suspected or confirmed influenza who is hospitalized, has severe or progressive illness, or is at high risk for complications from influenza, even if the illness seems mild. Persons at high risk include young children (especially children aged <2 years), pregnant women, persons with chronic medical conditions like asthma, diabetes, or heart disease, and adults aged ≥65 years.

Ideally, antiviral treatment should be initiated within 48 hours of symptom onset, when treatment is most effective (4). However, antiviral treatment initiated later than 48 hours after illness onset can still be beneficial for some patients. Observational studies of hospitalized patients suggest some benefit when treatment was initiated up to 4 or 5 days after symptom onset (4). Also, a randomized placebo-controlled study suggested clinical benefit when oseltamivir was initiated 72 hours after illness onset among febrile children with uncomplicated influenza (6). Clinical judgment, on the basis of the patient’s disease severity and progression, age, underlying medical conditions, likelihood of influenza, and time since onset of symptoms, is important when making antiviral treatment decisions for outpatients. The decision to initiate antiviral treatment should not be delayed pending laboratory confirmation of influenza, especially if performed by insensitive assays, such as rapid influenza diagnostic tests. Health care providers should advise patients at high risk to call promptly if they get symptoms of influenza. Also, clinicians should have a high index of suspicion for influenza while influenza activity is widespread. Alternative strategies, such as health care provider–operated telephone triage, might enable patients at high risk to discuss symptoms over the phone and facilitate early initiation of treatment.

What is already known on this topic?

Effectiveness of seasonal influenza vaccine can vary and depends in part on the match between vaccine viruses and circulating influenza viruses. However, influenza vaccination, even with low effectiveness, prevents thousands of hospitalizations.

What is added by this report?

So far this season, more than two thirds of influenza A (H3N2) viruses are different from the H3N2 component of 2014–15 influenza vaccine. Based on data from 2,321 children and adults with acute respiratory illness enrolled during November 10, 2014–January 2, 2015, at five study sites with outpatient medical facilities in the United States, the overall estimated effectiveness of the 2014–15 seasonal influenza vaccine for preventing medically attended, laboratory-confirmed influenza virus infection was 23%.

What are the implications for public health practice?

Early estimates indicate that influenza vaccines provide limited protection against influenza viruses circulating so far during 2014–15 season, which were mainly influenza A (H3N2) viruses. Although vaccination should continue as long as influenza viruses are circulating, treatment with influenza antiviral medications is more important than usual. All hospitalized patients and all outpatients at high risk for serious complications should be treated as soon as possible with one of three available influenza antiviral medications if influenza is suspected.

Although antigenic match influences vaccine effectiveness, randomized studies of influenza vaccines have reported variable vaccine efficacy during seasons when antigenically drifted viruses predominated (7). Since October 1, 2014, drifted influenza A (H3N2) viruses have accounted for an increasing proportion of antigenically characterized A (H3N2) isolates relative to A/Texas/20/2012-like viruses (8). Drifted A (H3N2) viruses were first identified in a small proportion of surveillance specimens in late March 2014, after the World Health Organizations had selected the strains for inclusion in the 2014–15 Northern Hemisphere vaccine. These antigenically drifted viruses were detected with increasing frequency from July to September 2014, when they had become common among A (H3N2) viruses in the United States and abroad (9). As of January 3, 2015, 68% of A (H3N2) viruses isolated in the United States since October 1, 2014, were antigenically or genetically different from the A (H3N2) vaccine virus component (2); characterization of a limited number of A (H3N2) viruses from US Flu VE network enrollees had similar findings. Modeling conducted by CDC suggested that a VE of only 10% in older adults could prevent approximately 13,000 influenza-associated hospitalizations in adults aged ≥65 years in the United States during a moderately severe influenza season such as the 2012–13 influenza season (3). Vaccination is particularly important for persons at high risk for serious influenza-related complications and their close contacts.

The findings in this report are subject to at least four limitations. First, these early VE estimates are imprecise for persons aged ≥18 years, limiting ability to detect statistically significant protection against influenza illness resulting in visits to health care providers; larger numbers of enrollees are required to detect significant protection when VE is low. Second, the VE estimates in this report are limited to the prevention of outpatient medical visits, rather than more severe illness outcomes, such as hospitalization or death; studies are being conducted during the 2014–15 season to estimate VE against more severe illness outcomes. Third, vaccination status included self-report at four of five sites, and dates of vaccination and vaccine formulation were available only for persons with documented vaccination obtained from medical records or immunization registries; complete vaccination data are needed to verify vaccination status and estimate VE for different vaccine formulations. Finally, future interim estimates and end-of-season VE estimates could differ from current estimates as additional patient data become available or if there is a change in circulating viruses late in the season.

Although influenza vaccines are the best tool for prevention of influenza currently available, more effective vaccines are needed. Other practices that can help decrease the spread of influenza include respiratory hygiene, cough etiquette, social distancing (e.g., staying home from work and school when ill or staying away from persons who are ill) and hand washing. Antiviral medications are an important adjunct in the treatment and control of influenza for the 2014–15 season and should be used as recommended, regardless of patient vaccination status.

## Figures and Tables

**TABLE 1:** Selected characteristics of enrolled patients with medically attended acute respiratory illness, by influenza test result status and seasonal influenza vaccination status — U.S. Influenza Vaccine Effectiveness Network, United States, November 10, 2014–January 2, 2015

Characteristic	Test result status					Vaccination status			
	
	Influenza positive		Influenza negative		p value†	Vaccinated*			p value†
		
	No.	(%)	No.	(%)		No.	Total	(%)	
**Overall**	**950**	**(41)**	**1,371**	**(59)**		**1,236**	**2,321**	**(53)**	
**Study site**					**<0.001**				**<0.001**
Michigan	202	(41)	286	(59)		258	488	(53)	
Pennsylvania	239	(52)	222	(48)		210	461	(46)	
Texas	210	(41)	297	(59)		252	507	(50)	
Washington	114	(24)	361	(76)		313	475	(66)	
Wisconsin	185	(47)	205	(53)		203	390	(52)	
**Sex**					**0.63**				**0.01**
Male	402	(40)	594	(60)		499	996	(50)	
Female	548	(41)	777	(59)		737	1325	(56)	
**Age group (yrs)**					**<0.001**				**<0.001**
6 mos–8	225	(35)	413	(65)		302	638	(47)	
9–17	185	(52)	170	(48)		142	355	(40)	
18–49	268	(40)	400	(60)		307	668	(46)	
50–64	136	(38)	223	(62)		240	359	(67)	
≥65	136	(45)	165	(55)		245	301	(81)	
**Race/Ethnicity** §					**0.02**				**<0.001**
White	713	(41)	1021	(59)		970	1734	(56)	
Black	89	(50)	90	(50)		61	179	(34)	
Other race	78	(37)	132	(63)		107	210	(51)	
Hispanic	66	(35)	123	(65)		95	189	(50)	
**Self-rated health status** ¶					**0.17**				**0.01**
Fair or poor	43	(38)	70	(62)		66	113	(58)	
Good	184	(37)	312	(63)		281	496	(57)	
Very good	328	(41)	474	(59)		442	802	(55)	
Excellent	388	(43)	514	(57)		443	902	(49)	
**Illness onset to enrollment (days)**					**<0.001**				**0.15**
<3	451	(56)	354	(44)		420	805	(52)	
3–4	330	(37)	553	(63)		458	883	(52)	
5–7	169	(27)	464	(73)		358	633	(57)	
**Influenza test result**									
Negative			1,371			771	1,371	(56)	
Influenza B positive**	35					17	35	(49)	
Influenza A positive**	916					448	916	(49)	
A (H1N1)pdm09	0					0	0	(0)	
A (H3N2)	842					407	842	(48)	
A subtype pending	74					41	74	(55)	

* Defined as having received ≥1 dose of vaccine ≥14 days before illness onset. A total of 92 participants who received the vaccine ≤13 days before illness onset were excluded from the study sample.

† The chi-square statistic was used to assess differences between the numbers of persons with influenza-negative and influenza-positive test results, in the distribution of enrolled patient and illness characteristics, and in differences between groups in the percentage vaccinated.

§ Enrollees were categorized into one of four mutually exclusive racial/ethnic populations: white, black, other race, and Hispanic. Persons identified as Hispanic might be of any race. Persons identified as white, black, or other race are non-Hispanic. The overall prevalences calculated included data from all racial/ethnic groups, not just the four included in this analysis. Race/ethnicity data were missing for nine enrollees.

¶ Data on self-rated health status were missing for eight enrollees.

** One patient had coinfection with influenza A (H3N2) and influenza B, making the sum 951, or one greater than the total number of influenza positives.

**TABLE 2:** Number and percentage receiving 2014–15 seasonal influenza vaccine among 2,321 outpatients with acute respiratory illness and cough, by influenza test result status, age group, and vaccine effectiveness* against all influenza A and B and against virus type A (H3N2) — U.S. Influenza Vaccine Effectiveness Network, United States, November 10, 2014–January 2, 2015

Influenza type/Age group	Influenza positive			Influenza negative			Vaccine effectiveness			

							Unadjusted		Adjusted	
			
	No. vaccinated	Total sample	(%)	No. vaccinated	Total sample	(%)	(%)	(95% CI)	(%)	(95% CI)
**Influenza A and B**										
**Overall**	**465**	**950**	**(49)**	**771**	**1,371**	**(56)**	**(25)**	**(12–37)**	**(23)**	**(8–36)**
Age group (yrs)										
6 mos–17	159	410	(39)	285	583	(49)	(34)	(14–49)	(24)	(0–43)
18–49	114	268	(43)	193	400	(48)	(21)	(−8–42)	(16)	(−18–41)
≥50	192	272	(71)	293	388	(76)	(22)	(−10–45)	(23)	(−14–47)
**Influenza A (H3N2)**										
**Overall**	**407**	**841**	**(48)**	**771**	**1,371**	**(56)**	**(27)**	**(13–39)**	**(22)**	**(5–35)**
Age group (yrs)										
6 mos–17	143	375	(38)	285	583	(49)	(35)	(16–50)	(26)	(2–45)
18–49	100	235	(43)	193	400	(48)	(21)	(−10–43)	(12)	(−26–39)
≥50	164	231	(71)	293	388	(76)	(21)	(−15–45)	(14)	(−31–43)

**Abbreviation:** CI = confidence interval.

* Vaccine effectiveness was estimated as 100% × (1 − odds ratio [ratio of odds of being vaccinated among outpatients with influenza-positive test results to the odds of being vaccinated among outpatients with influenza-negative test results]); odds ratios were estimated using logistic regression.

## Footnotes

* The U.S. Flu VE Network sites and the dates enrollment began were as follows: the University of Michigan School of Public Health (the University of Michigan School of Public Health, partnered with the University of Michigan Health System, Ann Arbor, and the Henry Ford Health System, Detroit, Michigan) (November 10, 2014); Baylor Scott and White Health, Texas A&M University Health Sciences Center College of Medicine (Temple, Texas) (November 13, 2014); Group Health Cooperative (Seattle, Washington) (December 1, 2014); the University of Pittsburgh Schools of the Health Sciences and UPMC (Pittsburgh, Pennsylvania) (November 24, 2014); and the Marshfield Clinic Research Foundation (Marshfield, Wisconsin) (December 8, 2014).

† Influenza vaccination coverage estimates for the 2014–15 season are available at http://www.cdc.gov/flu/fluvaxview/index.htm.

§ Groups at higher risk for influenza complications include 1) children aged <5 years (especially those aged <2 years); 2) adults aged ≥65 years; 3) persons with chronic pulmonary conditions (including asthma); cardiovascular disease (except hypertension alone); renal, hepatic, or hematologic (including sickle cell) disease; metabolic disorders (including diabetes mellitus); or neurologic and neurodevelopmental conditions (including disorders of the brain, spinal cord, peripheral nerve, and muscle, such as cerebral palsy, epilepsy [seizure disorders], stroke, intellectual disability [mental retardation], moderate to severe developmental delay, muscular dystrophy, and spinal cord injury); 4) persons with immunosuppression, including that caused by medications or by human immunodeficiency virus infection; 5) women who are pregnant or postpartum (within 2 weeks of delivery); 6) persons aged ≤18 years who are receiving long-term aspirin therapy; 7) American Indians/Alaska Natives; 8) persons who are morbidly obese (i.e., body mass index ≥40); and 9) residents of nursing homes and other chronic care facilities.

¶ A complete summary of guidance for antiviral use is available at http://www.cdc.gov/flu/professionals/antivirals/summary-clinicians.htm.
